# Cytokine profiles in nasal fluid of patients with seasonal or persistent allergic rhinitis

**DOI:** 10.1186/s13223-015-0093-x

**Published:** 2015-09-22

**Authors:** Katrin König, Christine Klemens, Katharina Eder, Marion San Nicoló, Sven Becker, Matthias F. Kramer, Moritz Gröger

**Affiliations:** Department of Otorhinolaryngology, Head and Neck Surgery, University Medical Center Großhadern of the Ludwig-Maximilians-University Munich, Marchioninistr. 15, 81377 Munich, Germany; Department of Otorhinolaryngology, Head and Neck Surgery, University Medical Center of the Johannes Gutenberg University Mainz, Langenbeckstr. 1, 55101 Mainz, Germany

**Keywords:** Allergic rhinitis, Nasal secretion, Mediators, Cytokines, Chemokines, Interleukins

## Abstract

**Background:**

New therapeutic approaches with biologic agents such as anti-cytokine antibodies are currently on trial for the treatment of asthma, rhinosinusitis or allergic diseases necessitating patient selection by biomarkers. Allergic rhinitis (AR), affecting about 20 % of the Canadian population, is an inflammatory disease characterised by a disequilibrium of T-lymphocytes and tissue eosinophilia. Aim of the present study was to describe distinct cytokine patterns in nasal secretion between seasonal and perennial AR (SAR/PAR), and healthy controls by comparing cytokines regulating T-cells or stimulating inflammatory cells, and chemokines.

**Methods:**

Nasal secretions of 44 participants suffering from SAR, 45 participants with PAR and 48 healthy controls were gained using the cotton wool method, and analysed for IL-1β, IL-4, IL-5, IL-6, IL-10, IL-12, IL-13, IL-17, GM-CSF, G-CSF, IFN-γ, MCP-1, MIP-1α, MIP-1β, eotaxin, and RANTES by Bio-Plex Cytokine Assay as well as for ECP and tryptase by UniCAP-FEIA.

**Results:**

Participants with SAR or PAR presented elevated levels of tryptase, ECP, MCP-1, and MIP-1β, while values of GM-CSF, G-CSF, IL-1β, and IL-6 did not differ from the controls. Increased levels of IL-5, eotaxin, MIP-1α, and IL-17 and decreased levels of IFN-γ, IL-12 and IL-10 were found in SAR only. RANTES was elevated in SAR in comparison to PAR. Interestingly, we found reduced levels of IL-4 in PAR and of IL-13 in SAR.

**Conclusions:**

Elevated levels of proinflammatory cytokines were seen in both disease entities. They were, however, more pronounced in SAR, indicating a higher degree of inflammation. This study suggests a downregulation of T_H_1 and T_reg_-lymphocytes and an upregulation of T_H_17 in SAR. Moreover, the results display a prominent role of eosinophils and mast cells in AR. The observed distinct cytokine profiles in nasal secretion may prove useful as a diagnostic tool helping to match patients to antibody therapies.

## Background

Allergic rhinitis (AR) is a common disorder of the nose. Patients’ symptoms include nasal obstruction, rhinorrhoea, sneezing and nasal itching. All of them are reversible spontaneously or under treatment. AR is subdivided into intermittent and persistent disease. Intermittent disease is defined by the patient having symptoms for less than 4 days a week or for less than 4 weeks [1]. It is estimated that 400 million people worldwide are affected, with a prevalence of AR of about 20 % in Canada and 23 % in Europe [2–4]. Todo-Bom et al. [5] found that intermittent and persistent disease are equally frequent in adults. AR is often associated with asthma, sinusitis, otitis media or nasal polyps and has a significant impact on patients’ quality of life [1, 6]. In addition, the disease imposes a substantial economic burden for society [7].

The underlying pathology of AR is known to be a type 1 immediate hypersensitivity reaction. During the period of sensitisation, the allergen is presented to CD4+ T-lymphocytes inducing differentiation to the T-helper cell (T_H_) 2 phenotype. T_H_2-lymphocytes secrete cytokines which promote the differentiation of B cells as well as induce immunoglobulin (Ig) synthesis and regulate Ig isotype switching. This results in increased levels of specific IgE, both local and systemic [8]. In the early-phase of allergic reaction, mast cells, coated with specific IgE, recognise the allergen and release several mediators such as histamine and tryptase. In contrast, the late-phase is characterised by the secretion of chemokines like eosinophil chemotactic protein (eotaxin), “regulated on activation, normal T cell expressed and secreted” (RANTES), and macrophage inflammatory protein-1α (MIP-1α) [9], which induce the recruitment of eosinophils and other inflammatory cells. Activated eosinophils release granules containing amongst others eosinophil cationic protein (ECP) and major basic protein (MBP) [10]. In addition, eosinophils synthesise and secrete cytokines, e.g. interleukin (IL)-5 or granulocyte–macrophage colony-stimulating factor (GM-CSF). Whereas the early-phase response to allergen exposure leads to acute symptoms, the late-phase reaction is held responsible for persisting inflammation.

AR is determined by a disequilibrium of T-helper cells with a predominance of T_H_2-type cytokines but normal levels of T_H_1-type cytokines. Another subtype of T-cells, regulatory T-cells (T_reg_), suppresses both T_H_1 and T_H_2-type cytokine expression [11]. Thus, it has been suggested that in AR, an imbalance between T_H_2 and T_reg_-cells exists as well [10]. Concerning T_H_17-lymphocytes, some authors found elevated levels of IL-17. However, the findings on IL-17 are ambiguous and the role of T_H_17-cells in AR remains unclear [12, 13].

Aim of the present study was to investigate whether in AR caused by a seasonal (SAR) or a perennial (PAR) allergen, representative cytokines and mediators in nasal discharge show distinct patterns picturing the pathophysiology. Therefore, we analysed the levels of cytokines and other inflammatory mediators in the nasal fluid of participants suffering from SAR or PAR, focusing on three main topics: cytokines (1) regulating T_H_1 (interferon-γ (IFN-γ), IL-12), T_H_2 (IL-4, IL-13), T_reg_ (IL-10), and T_H_17 (IL-17) cells, or (2) stimulating and activating inflammatory cells like granulocytes and mast cells (granulocyte colony-stimulating factor (G-CSF), GM-CSF, IL-1β, IL-5, and IL-6), and (3) chemokines such as eotaxin, RANTES, monocyte chemotactic protein-1 (MCP-1), or MIP-1α/β.

## Methods

### Study population

Clinical history was taken by one of the investigators. Patients presenting a history of chronic rhinosinusitis, nasal polyposis or aspirin sensitivity were excluded from the study (Table 1). Any medication concerning the nasal disease during 6 weeks prior to the examination constituted an exclusion criterion, especially anti-inflammatory medication such as nasal steroids or antihistamines. Also, nasal endoscopy was performed in all participants in order to assess clinical signs of rhinitis and to exclude patients with signs of purulent rhinitis or polyposis. After exclusion, 137 volunteers (73 males, 64 females, mean age 38 ± 16 years) participated in this study.

**Table 1:** Exclusion criteria

All groups	Chronic rhinosinusitis Nasal polyposis Aspirin sensitivity Purulent rhinitis Specific medication during the last 6 weeks
SAR	Sensitisation to perineal allergen
PAR	Sensitisation to seasonal allergen

AR was determined by the participant’s history and by a positive skin prick test (SPT) (ALK-Abelló, Wedel, Germany) for the following allergens: timothy grass, rye, birch, hazel, alder, beech, mugwort, ribwort, nettle, dandelion, house dust mite, storage mite, dog, cat and horse epithelial dander, alternaria, aspergillus, cladosporium, and penicillium; histamine dihydrochloride solution at 1 mg/ml as positive control and allergen-free saline solution as negative control were used. The SPT was constituted positive if the diameter of the wheal was >3 mm. Thereafter, specific IgE to allergens tested positive in skin prick test was measured in serum (UniCAP-FEIA, Phadia, Freiburg, Germany).

SAR (n = 44) was determined by sensitisation to at least one seasonal allergen with a positive skin prick test and a compatible positive specific IgE measurement (≥0.8 kU/l) as well as typical seasonal complaints in participant’s history. If patient’s history did not allow a definite rating of the seen sensitisation with respect to its clinical relevance, a intranasal challenge to the suspected allergen was performed. Participants additionally sensitised to a perennial allergen were excluded.

PAR (n = 45) was determined by participant’s history, a sensitisation to house dust mite, animal dander, or perennial mold like aspergillus with a positive skin prick test and a specific IgE ≥ 0.8 kU/l. Moreover, an intranasal allergen challenge was performed in case of a sensitisation to house dust mite or perennial mold, or a sensitisation to animal dander whose clinical relevance could not be clearly rated by patient’s history. Participants additionally sensitised to a seasonal allergen were excluded.

Healthy controls (n = 48) presented no history of inflammatory nasal complaints and a negative in vitro allergy screening test Sx1 (Phadia, Freiburg, Germany).

Samples were collected during as well as outside pollen season. Collection was not done in relation to actual allergen exposure or actual complaints.

The study was approved by the ethics committee of the medical faculty of Ludwig-Maximilians-University and written informed consent was obtained from all participants.

### Biochemical and immunological methods

For sampling of nasal fluids, the cotton wool method was performed with minor modifications as invented by Rasp and coworkers [14]. Nasal secretions were gained as previously described using small cone-shaped cotton wool pieces (absorbent cotton, Hartmann, Heidenheim/Brenz, Germany) with a length of about 3 cm and a diameter of about 6 mm [15]. Introduced into the middle meatus of the nose, the cotton wool pieces were left in place for 20 min and were subsequently centrifuged (+4 °C, 2000*g*) on a sieve for 10 min [16].

Because of partially small volumes, all samples were diluted 1:5 and were analysed for IL-1β, IL-4, IL-5, IL-6, IL-10, IL-12, IL-13, IL-17, GM-CSF, G-CSF, IFN-γ, MCP-1, MIP-1α, MIP-1β, eotaxin, and RANTES using a human cytokine 17-plex panel (Bio-Plex Cytokine Assay, Bio-Rad Laboratories, Hercules, California). The cytokine assay uses fluorescently-addressed polystyrene beads with conjugated capture antibodies directed to the above-mentioned cytokines. After washing, a fluorescently marked detection antibody builds an immunoassay with the cytokine. For analysis, two lasers excite the fluorochromes: one for classifying each bead, the other for quantifying the amount of analyte bound [17]. Detection levels were 0.5 pg/ml.

ECP and tryptase were measured by ELISA (UniCAP-FEIA, Phadia, Freiburg, Germany). Thresholds for detection were 10 ng/ml for ECP and 5 ng/ml for tryptase.

### Statistics

SigmaPlot for Windows version 11.0 software (Systat Software, San José, California, USA) was used for statistical evaluation and graphical presentation. All data failed normality testing (Shapiro–Wilk). Therefore, the Kruskal–Wallis One Way Analysis of Variance (ANOVA) on Ranks was used for testing a statistically significant difference in the median values among the three groups. To isolate the group or groups that differ from the others, the All Pairwise Multiple Comparison Procedures (Dunn’s Method) was used in the following step. *p* values <0.05 were regarded as significant. For graphic presentation of results, data is given in a box plot with the median (horizontal line within the box), the 25th and 75th percentile (boundary of the box), and the 10th and 90th percentile (whiskers above and below the box). Significances are graphically represented between the corresponding plots: * indicates *p* value <0.05, ** *p* value <0.01, and *** *p* value <0.001.

## Results

44 participants suffering from SAR, 45 participants suffering from PAR and 48 healthy subjects were included in this study. Demographics and sensitisation profiles are depicted in Table 2. The mean age varied from 36 to 40 years. The highest percentage of subjects suffering from asthma was found in the SAR group, followed by the PAR group and the controls. Participants suffering from SAR were frequently sensitised to grass and birch while house dust mite and animal dander were the main antigens in PAR. In SAR as well as in PAR one participant (2 %) was sensitised to mold with alternaria (seasonal) or aspergillus (perennial) being the relevant allergen.

**Table 2:** Demographic data and results of specific IgE

	Controls	SAR	PAR
Participants (N)	48	44	45
Mean age (years)	40	37	36
Gender ♀/♂ (%)	62/38	34/66	42/58
Asthma (%)	9	24	18
Poaceae (%)	n.d.	83	0
Betulaceae (%)	n.d.	52	0
Asteraceae (%)	n.d.	12	0
House dust mite (%)	n.d.	0	82
Mold (%)	n.d.	2	2
Animal dander (%)	n.d.	0	27

*n.d.* not determined

**Fig. 1:**
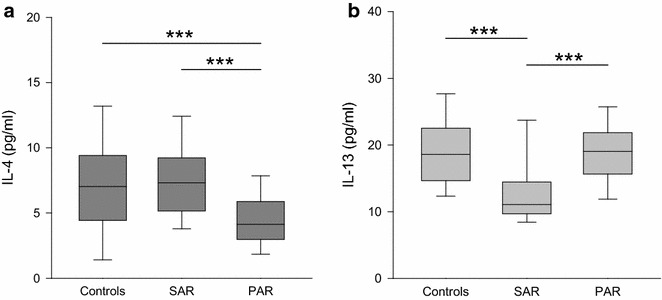
Levels of IL-4 and IL-13 in nasal fluid in controls, SAR and PAR: *box plots* of the levels of IL-4 (**a** *dark grey*) and IL-13 (**b** *light grey*) in nasal secretion are shown. IL-4 is significantly decreased in PAR compared to the controls as well as the SAR group. IL-13 is significantly decreased in SAR compared to both the controls and PAR. ***p < 0.001

AR is a T_H_2 dominated disease. Therefore, an increase of T_H_2 cytokines and possibly a decrease of T_H_1 and T_reg_ cytokines could be expected. Concerning the markers of T_H_2 induced B cell stimulation, we did not find elevated levels of either IL-4 nor IL-13. As shown in Fig. 1a, similar levels of IL-4 were found in SAR (median 7 pg/ml, range 2–17 pg/ml) and controls (median 7 pg/ml, range 0–32 pg/ml), but significantly lower levels in PAR (median 4 pg/ml, range 0–38 pg/ml) compared to controls as well as to SAR (*p* < 0.001 vs. controls/SAR). The quantity of IL-13 was decreased in SAR (median 11 pg/ml, range 6–137 pg/ml) compared to both the controls (median 19 pg/ml, range 10–32 pg/ml; *p* < 0.001) and PAR (median 19 pg/ml, range 7–48 pg/ml; *p* < 0.001) (Fig. 1b).

**Fig. 2:**
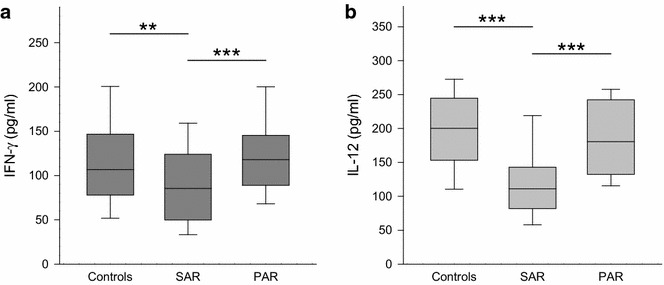
Levels of IFN-γ and IL-12 in nasal fluid in controls, SAR and PAR: *box plots* of the levels of IFN-γ (**a** *dark grey*) and IL-12 (**b** *light grey*) in nasal secretion are shown. IFN-γ is significantly decreased in SAR compared to the controls or PAR. IL-12 is significantly decreased in SAR compared to the controls as well as to PAR. **p < 0.01; ***p < 0.001

As pictured in Fig. 2a, b, a decrease of the T_H_1 marker cytokines IFN-γ and IL-12 was found in SAR (IFN-γ: median 85 pg/ml, range 5–299 pg/ml; *p* < 0.01 vs. control, *p* < 0.001 vs. PAR; and IL-12: median 111 pg/ml, range 45–299 pg/ml; *p* < 0.001 vs. control/PAR) compared to PAR (IFN-γ: median 118 pg/ml, range 18–822 pg/ml; and IL-12: median 180 pg/ml, range 71–348 pg/ml) and the controls (IFN-γ: 107 pg/ml, range 34–551 pg/ml; and IL-12: median 200 pg/ml, range 59–358 pg/ml).

**Fig. 3:**
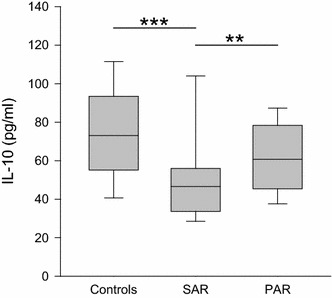
Levels IL-10 in nasal fluid in controls, SAR and PAR: *box plot* of IL-10 levels in nasal secretion is shown. IL-10 is significantly decreased in SAR compared to the controls as well as to PAR. **p < 0.01; ***p < 0.001

Moreover, the quantity of the mainly T_reg_ cell released cytokine IL-10 was lower in SAR (median 47 pg/ml, range 21–139 pg/ml) than in the controls (median 73 pg/ml, range 31–158 pg/ml; *p* < 0.001) and PAR (median 61 pg/ml, range 21–118 pg/ml; *p* < 0.01) (Fig. 3).

**Fig. 4:**
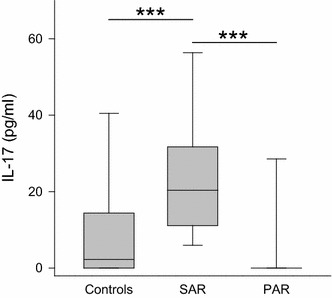
Levels IL-17 in nasal fluid in controls, SAR and PAR: *box plot* of IL-17 levels in nasal secretion is shown. IL-17 is significantly increased in SAR compared to both the controls and PAR. ***p < 0.001

IL-17 levels, representing T_H_17 activity, were significantly elevated in the SAR group (median 20 pg/ml, range 0–90 pg/ml; *p* < 0.001 vs. control/PAR) while the PAR group and the controls showed similar low levels (PAR: median 0 pg/ml, range 0–147 pg/ml; controls: median 2 pg/ml, range 0–320 pg/ml) (Fig. 4).

**Fig. 5:**
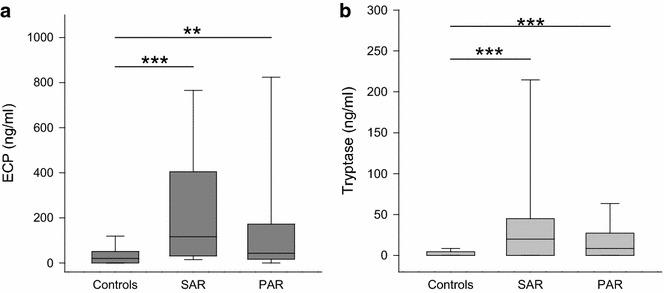
Levels of ECP and tryptase in nasal fluid in controls, SAR and PAR: *box plots* of the levels of ECP (**a** *dark grey*) and tryptase (**b** *light grey*) in nasal secretion are shown. ECP is significantly elevated in SAR and PAR compared to controls. Tryptase is significantly elevated in SAR and PAR compared to controls. **p < 0.01; ***p < 0.001

Investigating the stimulation and activation of inflammatory cells, several degranulation products and cytokines were measured. Depicted in Fig. 5a, a comparison of the levels of ECP as a marker of eosinophil activation in nasal mucosa revealed an increase in SAR (median 116 ng/ml, range 0–1000 ng/ml; *p* < 0.001) and PAR (median 43 ng/ml, range 0–1000 ng/ml; *p* < 0.01) compared to the controls (median 20 ng/ml, range 0–467 ng/ml). Likewise, tryptase levels displaying mast cell activation were significantly elevated in the nasal secretions of the SAR (median 20 ng/ml, range 0–452 ng/ml; *p* < 0.001) and the PAR group (median 9 ng/ml, range 0–1000 ng/ml, *p* < 0.001) compared to controls (median 0 ng/ml, range 0–94 ng/ml) (Fig. 5b). As shown in Table 3, for G-CSF and GM-CSF, no significant differences among the three groups were found. Also, the amount of IL-1β in the nasal secretions was rather similar in all groups. Levels of IL-5 in SAR were significantly increased over the controls. However, no statistically significant difference between the controls and PAR was seen. The measurement of IL-6 revealed no differences among the three groups.

**Table 3:** Cytokine levels in nasal fluid in healthy controls, SAR and PAR patients

	IL-1β	IL-5	IL-6	G-CSF	GM-CSF	Eotaxin	RANTES	MCP-1	MIP-1α
Controls	20	5	25	90	32	45	9	66	0
	4–1000	1–238	0–3036	9–7962	0–137	0–154	0–259	17–401	0–113
SAR	33	13	39	165	28	67	16	94	8
	2–1677	0–829	5–443	10–10,681	0–115	0–503	0–766	30–600	0–66
PAR	31	6	32	146	27	30	0	93	0
	5–7894	1–761	0–397	0–17,211	0–149	0–220	0–509	0–866	0–119
*p* values									
SAR-Con	n.s.	<0.05	n.s.	n.s.	n.s.	n.s.	n.s.	<0.01	<0.001
PAR-Con	n.s.	n.s.	n.s.	n.s.	n.s.	n.s.	n.s.	<0.05	n.s.
SAR-PAR	n.s.	n.s.	n.s.	n.s.	n.s.	<0.001	<0.01	n.s.	<0.001

Concentrations are given in pg/ml. Data are presented as median (upper line) and range (lower line)

*n.s.* not significant

**Fig. 6:**
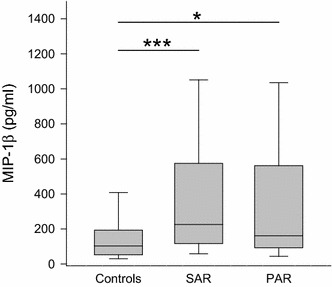
Levels of MIP-1β in nasal fluid in controls, SAR and PAR: *box plot* of MIP-1β levels in nasal secretion. MIP-1β is significantly elevated in SAR as well as in PAR compared to controls. *p < 0.05; ***p < 0.001

Also displayed in Table 3 are the levels of chemokines in nasal discharge of AR participants and controls. An elevation of eotaxin was found in SAR compared to PAR. Concerning RANTES, higher levels were detected in SAR than in PAR whereas no significant difference could be seen between the control group and either of the AR groups. In comparison to the controls, elevated levels of MCP-1 were found in both AR groups. MIP-1α showed a significantly elevated level in the SAR group compared to control as to PAR. For MIP-1β, compared to control (median 103 pg/ml, range 0–2049 pg/ml), an increase was found in SAR (median 226 pg/ml, range 16–1769 pg/ml; *p* < 0.001) as well as in PAR (median 161 pg/ml, range 0–2138 pg/ml; *p* < 0.05) (Fig. 6).

## Discussion

Nasal secretion is easily accessible and Bio-Plex Cytokine Assay is simple to perform. Thus, it constitutes a methodological approach possibly applicable in clinical routine. Cytokines in the nasal fluid of participants suffering from SAR or PAR were analysed in a true-to-life clinical setting. Aim of the present study was to investigate whether in AR, representative cytokines in nasal discharge show distinct patterns proving the used methodology helpful for endotyping of inflammatory nasal diseases.

For a lifelike approach, we chose to collect the samples neither during specific seasons of the year nor after allergen provocation. In SAR, the participants’ exposition to aeroallergens depends not only on the absolute amount of antigens in the air but also on the habitation, profession and habits of the individual participant as well as his efforts of abstention. Likewise, it is difficult to find objective measurements for the individual pollination in PAR participants’ everyday life which also varies in the course of the year [18]. We thus refrained from determining the exact pollution with antigens. Moreover, not using subjective or objective measures of AR, we did not know if participants were actually suffering from AR at the time of sample collection. The magnitude of the allergic response is associated with the preseasonal values of IgE [8] and the levels of cytokines were found to differ between atopic and non-atopic subjects during as well as outside the pollen season [19]. Addressing the important question of trends in cytokine levels over time, longitudinal studies instead of the presented cross-sectional study are mandatory.

IL-4 and IL-13 are produced by T_H_2-cells and other inflammatory cells such as mast cells, eosinophils or basophils [20]. In the pathology of allergy, similar responses to these cytokines are known. They act in concert or alone to induce differentiation of T_H_-cells, migration of T-cells and eosinophils, Ig class switching or mucus secretion [20, 21]. In the present study, we surprisingly found normal or decreased levels of these T_H_2 characterising cytokines, contradicting an expected upregulation, which would lead to stimulation of IgE production. Previous studies on IL-4 and IL-13 revealed normal or elevated levels in nasal secretions under natural allergen exposure, while increases were reported after provocation tests [9, 15, 19, 22]. One group found decreased levels of IL-4 in SAR patients [23]. We measured the cytokine levels without prior nasal allergen challenge, which might explain the missing elevations in our study. On the one hand it might be concluded that the amount of allergens in natural environment is not high enough to provoke profuse production of IL-4 and IL-13 but on the other hand this cannot explain decrease. No definite explanation can be given to the normal or even decreased values of IL-4 and IL-13, a methodological cause cannot be ruled out.

Although allergy is known to be a T_H_2-dominated disease, the role of other T-cell subsets was also of interest in the presented work. IL-12 and IFN-γ are well-known indicators of T_H_1-type inflammation and crucial to induction and maintenance of T_H_1 response, activating preferably phagocytic and cytotoxic immune cells [24, 25]. Moreover, these cytokines counteract the differentiation of T_H_2 and T_H_17 [24]. In our study, the levels of IL-12 and IFN-γ were both decreased in SAR but not in PAR. This points to a downregulation of T_H_1-lymphocytes in SAR.

Having the ability to reduce Ig production and tissue eosinophilia as well as T_H_2- and T_H_17-dependent reactions, regulatory T-cells are essential in maintaining peripheral tolerance. Allergen-specific T_reg_-cells have been reported to be diminished in PAR and have decreased suppressive capacity in SAR [26]. IL-10 is an immunomodulatory cytokine which, together with tumor growth factor (TGF)-β, is important for T_reg_ operability. Previous studies on the levels of IL-10 revealed discordant data. Unchanged or increased levels were found in naïve nasal secretions of SAR patients, while IL-10 was elevated after allergen provocation and specific immunotherapy [12, 22, 26]. Our results might suggest a diminished influence of T_reg_-cells in SAR, illustrating the impaired peripheral tolerance in AR. However, no final conclusion on T_reg_ can be drawn based on our results as IL-10 is produced by other cell types like T_H_2 cells as well.

IL-17 is a cytokine with proinflammatory properties influencing diverse cells. IL-17 producing cells, named T_H_17, were discovered in the beginning of this century. Though T_H_17-lymphocytes were a subject of interest in recent years, their role in AR remains unclear. Scadding suspects elevated levels of this cytokine, predominantly in PAR [12]. Our study does not support this thesis, showing an elevation of IL-17 in SAR but undetectable levels in the majority of the PAR samples. This is in line with a previous study reporting no elevation of IL-17 in nasal discharge of PAR patients [15].

Concerning the role of the discussed T-cell subsets in AR, our results suggest a downregulation of T_H_1 and T_reg_-lymphocytes especially in SAR. This indicates an imbalance between the different T-cell subsets resulting in an impaired tolerance to allergens. Furthermore, increased markers of T_H_17 activity were found in AR leaving this T-cell subset as a field of future research.

Mast cells, activated by antigen and IgE, immediately release preformed mediators such as histamine, serotonin, and tryptase. Moreover, stimulated mast cells newly produce a number of other mediators, which are released during the late-phase of allergic reaction [27]. We utilised tryptase as a marker of mast cell activation and detected elevated levels in both SAR and PAR. This is concordant with previous reports of elevated levels of tryptase caused by natural or artificial allergen exposure [28, 29] and emphasises the importance of mast cells in AR.

As the eosinophil is one of the predominant cell types in AR, we measured two indicators of eosinophil activation, ECP and IL-5. The level of ECP, which is secreted by eosinophils and important in the defence of pathogens, correlates positively with the number of nasal eosinophils [14, 30]. Consistent with previous reports, our study found significantly elevated levels of ECP [14]. The amount of nasal ECP was sixfold higher in SAR and doubled in PAR in comparison to the controls. IL-5, primarily produced by mast cells and T_H_2-lymphocytes, is thought to be responsible for eosinophil survival, chemotaxis, and activation [31]. This makes this cytokine a second suitable indicator of eosinophil activation. Just as for ECP, we found elevated levels in SAR, highlighting the importance of eosinophils in this disease entity. However, the concentration of IL-5 in PAR was in normal range. We conclude that the role of eosinophils might be less in PAR than in SAR, and other factors are more important in maintaining the more chronic inflammation. The elevated level of IL-5 in SAR might also be a possible therapeutic target. Pavord et al. [32] found reduced numbers of eosinophils in blood samples of asthmatics treated with an monoclonal antibody against IL-5. In conclusion, we found elevated levels of markers of eosinophil activation in both AR groups. However, the effect was more pronounced in SAR, suggesting a greater influence of eosinophils in SAR than in PAR.

Concerning the next group of cytokines, the colony-stimulating factors, surprisingly no increases could be shown. GM-CSF is a multifunctional proinflammatory cytokine produced by a host of different cells, amongst them epithelial cells, mononuclear cells or eosinophils. It acts locally in the nose stimulating dendritic cells as well as neutrophils and eosinophils. Moreover, GM-CSF induces proliferation and differentiation of stem cells [33]. An increase of GM-CSF in AR patients was reported [9]. However, we could not reproduce this finding, which might be due to the fact that our samples were taken without prior allergen provocation. For G-CSF, we did not find a statistically significant increase in either of the groups as well. G-CSF is known to regulate proliferation of haematopoietic progenitor cells and to influence neutrophil function. As most groups did not examine the amount of G-CSF in AR, little is known about its impact on AR. Pelikan [34] found elevated levels in tears of SAR patients after allergen provocation, providing evidence for an influence of G-CSF in this disease entity. But further research is needed to define its role in allergy.

We also measured the amount of two well-established proinflammatory cytokines, IL-1β and IL-6. For both cytokines, no statistically significant difference between the three groups was found. IL-1β and IL-6 are rather unspecific markers of inflammation. Various inflammatory cells are able to produce these pleiotropic cytokines. Physiologically, IL-1β and IL-6 influence the growth and maturation of immune cells as well as haematopoiesis. Furthermore, they are involved in auto-inflammatory diseases and oncogenesis [35, 36]. Data on these two general markers of inflammation in nasal fluids are inconsistent [12]. Pelikan [34] did not find elevated levels of IL-6 in tears of allergic subjects. This is consistent with our results, but disagrees with an elevation of IL-1β and IL-6 found by others [9]. An explanation could be that we examined nasal secretions under natural allergen exposure while elevated levels were described after experimental allergen challenge.

The late-response of allergic reaction is characterised by the influx of inflammatory cells into the site of inflammation. In this process, chemokines play a crucial role. To measure the recruitment of eosinophils, we examined three chemokines potently attracting these cells: eotaxin, RANTES, and MIP-1α. For all three, the concentration in nasal secretions of SAR participants was increased. Eotaxin, a specific eosinophil attractant, was elevated in SAR over the PAR group. Our results affirm the findings of Chawes et al. [22], who found elevated levels of eotaxin in nasal secretions of SAR patients under natural allergen exposure. Moreover, an increase of eotaxin positive cells and eosinophils in nasal biopsies was reported after allergen provocation [37]. Concerning RANTES, there was a significant elevation in SAR over PAR, while the differences between either of the AR groups and the controls were not significant. RANTES is not only known to attract eosinophils but also to cause activation of eosinophils and basophils resulting in inflammatory mediator release [21]. Further, elevated levels were reported after nasal allergen challenge [9]. The levels of MIP-1α were significantly increased in SAR participants. MIP-1α is produced by a number of inflammatory cells and is able to attract granulocytes as well as to activate eosinophils, to stimulate T-cells and to regulate Ig production [21, 38]. It is reported to be elevated after nasal allergen challenge [9, 12]. Interestingly, this chemokine was not detectable in the majority of our controls or PAR participants, while in SAR, most participants had detectable levels of MIP-1α. In summary, our results show an increase of eosinophil attractants in SAR. This is in line with the elevated levels of ECP and IL-5, emphasising the prominent role of eosinophils in SAR, while the normal levels of IL-5 and just slightly elevated levels of ECP in PAR indicate a minor role of eosinophils in the chronic inflammation of PAR.

The levels of MCP-1 and MIP-1β were elevated in either of the AR groups. Increased MCP-1 and MIP-1β release has been reported under natural exposure as well as after allergen provocation in SAR subjects [12, 22]. MCP-1 potently attracts and activates monocytes and basophils, and recruits macrophages and neutrophils [38, 39]. Secreted by monocytes, natural killer cells and activated lymphocytes, MIP-1β recruits lymphocytes, natural killer cells and immature dendritic cells [40]. The elevation of these two chemokines clearly shows that in both SAR and PAR, a bunch of diverse inflammatory cells is recruited. Our results thus support the concept of minimal persistent inflammation in PAR [41]. This concept states a persistent infiltration of neutrophils under continuous low allergen exposure while eosinophils and mast cells have minor influence.

## Conclusions

Aim of our study was to find distinct cytokine profiles in nasal discharge of AR participants in a lifelike approach, which might be useful for diagnostic purposes. Evaluating our results, ECP, tryptase, MCP-1, and MIP-1β are suitable markers to differentiate AR participants from healthy subjects. Furthermore, in SAR eotaxin, MIP-1α, and IL-17 are elevated in comparison to both PAR participants as well as controls. In addition, reduced levels of IFN-γ and IL-10 are found. Moreover, SAR and PAR can be distinguished by the levels of RANTES. Even though some questions remain unanswered, we have demonstrated that the methodology used in this study could be developed into a diagnostic tool for “endotyping” of patients in daily clinical routine. If such an “endotyping” is feasible in nasal discharge, this method is superior to immunohistochemical analysis of nasal biopsy specimen because nasal discharge is easily accessible and collection is harmless to the patient. Further research is needed to describe the cytokine patterns in nasal fluid of pure CRS with or without nasal polyps followed by examinations of mixed forms of CRS and AR. In the long term, easily accessible biomarkers could help to match patients with innovative therapeutic approaches like anti-cytokine antibodies. Uncovering specific endotypes out of clinically similar phenotypes might result in a more targeted, individualised therapy beneficial to the patient.
